# Being friendly to the skin microbiome: Experimental assessment

**DOI:** 10.3389/frmbi.2022.1077151

**Published:** 2023-02-02

**Authors:** Alex van Belkum, Paola Lisotto, Walter Pirovano, Sebastien Mongiat, Amine Zorgani, Mathias Gempeler, Radhika Bongoni, Eline Klaassens

**Affiliations:** ^1^ BaseClear BV, Leiden, Netherlands; ^2^ DSM Nutritional Products, Personal Care, Basel, Switzerland; ^3^ S-Biomedic NV JLBS, Beerse, Belgium

**Keywords:** skin microbiome, bacterial species, colonization, chemical compounds, formulation

## Abstract

Both academia and dermatological and cosmetic industries have acknowledged that healthy skin microbiota contribute to overall skin integrity and well-being. This implies that formulations developed for personal care (skin, scalp, hair etc) or (medical and cosmetic) treatment need to be compatible with microbiota conservation or possibly even improvement. The various chemical and biological components and mixtures thereof intended for direct application to the skin should not extensively affect the qualitative and quantitative composition of the skin microbiota. A compound should promote beneficial microbes and inhibit pathogens. Compounds but also final products could be considered at least theoretically “microbiome friendly” while in some cases changes to the microbiota may even be considered beneficial. An important hurdle lies in the practical and methodological approaches to be used for defining microbiota inertia of compounds and formulations. Clear guidelines for assessing microbiome friendliness are lacking. We propose three testing concepts that may help to define microbiome friendliness based on the assessment of minimal microbiota perturbation and possibly elimination of potential pathogens. Methods to prove microbiome friendliness should ultimately be based upon (metagenomic rather than amplicon-based) next generation sequencing of naive versus compound- or final product-exposed skin microbiota *in vivo*, but preferably also including *in vitro* and *ex vivo* pre-screening methodologies to build an understanding of their consequences. As in many domains of microbiome research, the development of experimental process controls and internal standards, which are essentially lacking to date, should be taken as a future prerequisite. There is also a requirement from regulatory agencies to define and harmonize acceptance criteria.

## Introduction

The precise physiological functions of microbial communities are a challenge to decipher. Microbiota are affected by a multitude of factors including nutrition, personal care, housing and living conditions, medical treatment and many others (Bouslimani et al., 2015). A common requirement is that methods and strategies for microbiota conservation and monitoring are required (Sonnenburg and Sonnenburg, 2019). High-throughput microbial shotgun genome sequencing has shed light on the complexity of the microbiota colonizing the bodies of higher eukaryotes, mankind included (e.g. Saheb Kashaf et al., 2022). Catalogues with microbial species inhabiting various physiological niches have been developed over the past decades although none of these can be considered complete yet. Simultaneously, there have been numerous studies suggesting or even defining the protective effect of native bacterial or fungal microbiota in cases of infectious challenges (Bjerre et al., 2017; Byrd et al., 2018; Wong and Levy, 2019; Carrieri et al., 2021; Langdon et al., 2021). This has led to enhanced appreciation of the roles and functions that our microbiota play in maintaining overall health status. Consequently, local or systemic disruption or perturbation of “healthy” microbiota has been associated with a variety of diseases. There is, however, not a clear definition of what represents a “healthy” microbiota. We postulate that “healthy” microbiota are those of an individual without an overt dysbiosis or disease condition. “Healthy” microbiota can differ from one individual to another. We postulate that the most dominant microbial taxa are a largely constant factor and maintain similar distributions and relative abundance levels.

Our lifestyle and acquired beauty regimens have resulted in a great diversity of ingredients that are repetitively applied to our skin. This will alter the molecular and microbial diversity as well as their dynamics on our various skin types. This temporal variability is also person and product dependent. To limit the overall impact of beauty routines as much as possible, some cosmetic formulations may aim to be “skinimalistic”, meaning that they should not affect physical and physiological skin status (www.shape.com/lifestyle/beauty-style). This less-is-more concept also contributes to a lower risk of allergy development or other sensitization phenomena (Taïeb, 1999; Baurecht et al., 2018). In parallel there is increasing knowledge that the skin microbiota are physiologically and clinical protective and efforts should be made to limit a de-stabilizing effect on the skin microbiota for certain formulations (see for instance www.kindtobiome.com and Murphy et al., 2021). This, in turn, has led to an increasing interest among dermatological and cosmetic industries for products that help define their treatment compounds and final products and interventions as “microbiota-safe” or “microbiota-friendly” (McBain et al., 2019). There is significant commercial interest from within the industry to facilitate claims on positive effects of their products on the microbiome in general. Although there are several products that are visibly labeled as being microbiome friendly, there are no universally accepted guidelines nor regulations defining the exact criteria that help to substantiate these claims. The closest regulation in the space is the Commission Regulation (EU) No 655/2013 in Europe but this has been shown to be incomplete and up for improvement (e.g. Staton, 2021). In the EU, the safety of a cosmetic product must be demonstrated and data on microbiological quality must be included in the Cosmetic Product Safety Report (CPSR). This is part of the Product Information File (PIF). The total count of aerobic mesophilic microorganisms (bacteria, yeasts and molds) and the absence of specific microorganisms (*Candida albicans*, *Staphylococcus aureus*, *Pseudomonas aeruginosa*, *Escherichia coli*) should be included in the file. Again, this does not relate to any direct parameter of microbiome friendliness.

The skin microbiome composition is very dynamic between healthy individuals. The microbiome is variable and being reproduced in alternative ways as dependent on life stochasticity. This requires a systems approach to even begin to get a mechanistic understanding of the host organism in its native environment, where the skin includes the cutaneous immune system, the skin microbiome and commercial cosmetic and/or medical product. Changes in microbiome composition can be due to fundamental features such as diet, lifestyle, genetics and genetic disorders, state of the immune system and more. Understanding the systems biology principles, deciphers the most essential mechanisms behind the host-microbiome interactions (Byrd et al., 2018; Khmaladze et al., 2020). For adequate assessment of microbiome friendliness all these factors should be included. This is experimentally very hard to achieve.

Skin integrity should not suffer from extraneous compounds and the primary barrier function should be protected (Elias, 2007). This also involves maintaining a slightly acidic pH value and the local production of antimicrobial peptides. Abnormal or elevated desquamation should be prevented and local production of cytokines should remain balanced (Matsui and Amagai, 2015). This will help to stabilize the microbial ecosystem as well including the local production of microbial metabolites which can also contribute to skin cell viability. A microbiota-friendly environment needs to be promoted and maintained. We propose a simple and pragmatic protocol for better and standardized definition of skin microbiota friendliness. Ultimately this should benefit customers in making balanced decisions on which product to buy.

## Testing concepts

**Figure 1:**
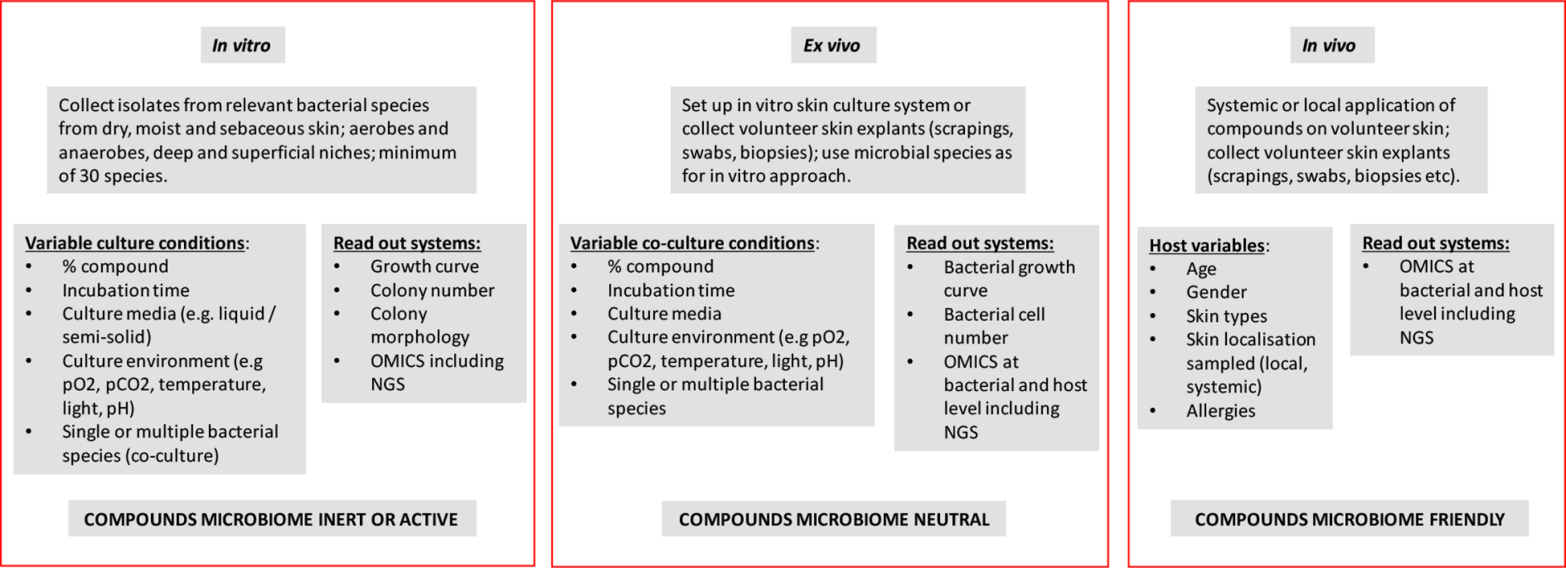
Schematic representation/flow sheet defining the experimentation needed for defining the microbiome neutral of friendly nature of (mixtures of) extracorporeal chemical and natural compounds.

We here define classical microbiological and molecular testing methods for the detection of changes in skin microbiota. The proposed tests are growth- or culture-based, but additional analytical methods also including next generation sequencing (NGS) have been described before (Van Belkum et al., 2018; Sfriso et al., 2020). NGS and qPCR can be targeted to specific microbial species or genera but can also be non-restricted and metagenomic in nature. Both methods have advantages but also disadvantages with regard to their comprehensiveness and completeness. Single species approaches are different from metagenomic approaches where taxonomic resolution reaches the strain level for multiple species simultaneously (Khachatryan et al., 2020). This higher taxonomic resolution will generate a more complete and far more detailed microbiota analyses for all kingdoms. We recommend shotgun metagenomics more than high-throughput amplicon sequencing (e.g. 16S rRNA genes or internal transcribe ribosomal spacers) as the key method for defining microbiome friendliness (**Figure 1**). This method will help tracing non-cultivable microbial species as well. NGS can be promoted given the continuing rise in affordability, high-quality data generation, speed and, hence, turn-around time.

Skin microbiota challenging consists of the application of a monomolecular compound, either in its pure form or at a given concentration in a (watery) solution or suspension. An effect exerted by a compound can also be construed as positive as in for instance the elimination of (a) pathogen(s) although in this manuscript we will not specifically address antibiotics and antiseptics. Using bacteriophage-derived lysins this approach has been shown successful in the elimination of *Staphylococcus aureus* (Eichenseher et al., 2022). The physiological state of the skin, frequency and duration of use, dilution or rinsing during use of the product and the location on the body can also be significant protective factors (Bouslimani et al., 2019; Murphy et al., 2021). The same line of thought can be followed for more complex (bio-)chemical compositions. Our premise is that if the effect of the individual compounds is known, then mixtures of those compounds and final products may have similar or even synergistic effects. This has to be calibrated and validated in follow up experiments, of course. It goes without saying that purity of the compounds and supplementation of the microbiome from deeper layers in the skin are important contributors to reproducibility of the studies proposed below. We will discuss three fundamentally different experimental testing models (*in vitro*, *ex vivo* and *in vivo*) and the preferred laboratory methodology will be listed (**Figure 1**).

### *In vitro* testing

The simplest testing model is one in which bacteria from the species that are most prevalent on dry, wet and/or sebaceous skin are directly exposed to the compound, mixes of compounds or final products in question. The microbial species involved are relatively well known and several are easy to cultivate aerobically without a need for complicated laboratory equipment (Byrd et al., 2018). Using liquid and semi-solid culture media that are as representative as possible for skin type and conditions, the effect of compounds can be assessed by classical detection of growth inhibition, colony-morphological changes or microbial killing. This is difficult in and of itself as the general purpose of culture media is to get microorganisms to grow, not mimic anything. With cultivation of skin specific organisms, the development of artificial sebum, for example, will be hard to include. Also, aerotolerant anaerobic bacterial species such as *Cutibacterium acnes* (formerly known as *Propionibacterium acnes*) are difficult to culture aerobically and in *in vitro* settings. Similar assays can be performed for mixtures of representative strains, where the complexity of the consortia can be simple (2-4 species) or more complex (up to more than 10 species in a single community) (Duncker et al., 2021). To add complexity to this, often the representative microbial cocktails for *in vitro* testing may cover different microbial kingdoms. Variables such as test duration, incubation temperature and composition of the culture medium and/or atmosphere can be included in the experimental design. Sampling can be done over time and in case of solid media also the changes in bacterial colony morphology can be documented by photography or videoing (Bär et al., 2020). Results of these tests will show whether or not, under these simple biological and ecological conditions and their variants, the components affect microbial growth parameters and other simple phenotypes. Read out systems can be kept as simple as sketched above but proteomic or transcriptomic approaches can be used to gather more detail (Pinto et al., 2020). Of considerable importance is also the discussion on the positive or negative effects of microbiota changes. Removal of opportunistic pathogens, for instance, leads to a clear change of microbiota and this should be interpreted as a positive rather than a negative effect (Chris Callewaert et al., 2021; Hill, 2021). A significant panel of putative pathogens should be studied in order to control for emergence of pathogens.

In case the components do show bacterial inhibition at any level during the *in vitro* testing it is recommended to further analyze the compound(s) for genuine antibacterial activity. In this respect, it would be good to develop guidelines for quantification of the effect. A 1-log reduction in the colony forming unit (CFU) count *in vitro* is of lesser importance than the killing of all cells but this may be different *in vivo*. To facilitate quantification, there are many technologies that can help to define minimum inhibitory concentrations for the compounds (Datar et al, 2021). Even the possible development of resistance to the compounds or final products among the key bacterial skin-inhabiting species should be investigated. Resistance development should be a strong counterindication for further use of the compound(s). If there are no clearly measurable effects then the compound “passes the test”. The more species tested, including those from deeper skin layers, the more comprehensive the results will be accurately representing the dynamics between aerobic and anaerobic microbiota.

### *Ex vivo* testing

The *in vitro* testing as described above can be repeated in the presence of cultured human skin cells and possibly even immune cells (Eichenseher et al., 2022). This can be done using skin cell lines in non-differentiated format or more differentiated ones using systems where the host cells grow at air-liquid interfaces. Host cells can be inoculated with individual bacterial species or mixtures thereof and environmental conditions can be modulated as described in the previous section. The first process control to be implemented is to control for the induction of a reaction from the skin cells themselves. If this test is negative, then comparing the artificial skin system behavior in the presence or absence of compounds will shed light on their effects on both microbial and host cells. This will provide a more complete picture than *in vitro* testing only, especially since here also stress in host cells can be measured (Cywes Bentley et al., 2005). Analytical methods can be similar to those used during the *in vitro* assessments.

Cultured skin cells, immortalized cell lines and/or host explants (cell scrapings, biopsies etc.) can be incubated in the presence or absence of the compounds and using culture driven and molecular testing formats the effect of compounds on resident microbiota can be assessed. For both host cell culture and explant studies especially the molecular and genomic data will allow for reliable decision making on compound inertia (Chua et al., 2022), better than compared to purely *in vitro* studies.

For the gastro-intestinal microbiota several model systems have been develop that more closely mimic the physiological situation and can be explored longitudinally for prolonged periods of time [e.g. Simulator of the Human Intestinal Microbial Ecosystem (SHIME) (Hernandez-Hernandez, 2019) and the Dutch TIM-1 and TIM-2 systems (Uriot et al., 2021)]. These are the first models where native microbiota can be inoculated in *in vitro* systems and maintained under a variety of modifiable conditions. The lack of similar systems in the context of skin is an area requiring further attention.

### *In vivo* testing

For *in vivo* testing, skin needs to be actively and sometimes invasively sampled, from healthy volunteers, before, during and after treatment with the compounds to be investigated. Sampling mostly consists of swabbing (dry or moist), tape stripping, scraping and the taking of punch biopsies. Sample processing and storage needs to be standardized as much as possible to assure study reproducibility (Ruuskanen et al., 2022). Sample quality may differ depending on prior skin treatments (showering, swimming, bathing, sauna, exposure to direct sunlight etc.) and guidance criteria designed to standardized these effects prior to sampling needs to be developed. *In vivo* testing can be done directly and *in situ* using experimental animals (which is impossible for purely cosmetic compounds due to current regulations that essentially block animal trials) or human volunteers. Direct application of compounds and/or final products on the skin and serial sampling of microbiota, defining the microbiome using high-density cultivation (culturomics, see Lewis et al., 2021) and NGS and performing detailed bioinformatic analyses will provide ultimate proof of compound effect on resident skin microbiota (Röttjers and Faust, 2022). Concentration of compounds and duration of their (repeated) application can be modulated and both short and long term effects can be assessed. *In vivo* testing is ethically most complicated and should be preceded by extensive toxicity and teratogenicity testing of the compounds involved. In case of cosmetic or therapeutic admission activities this is the most appropriate way of defining microbiota friendliness, although the comprehensiveness of *in vivo* testing strongly depends on the panel of volunteers involved (e.g. McBain et al., 2019). *In vivo* testing may lead to outcomes confounded by significant intrinsic differences between individuals. Outcomes may be dependent on the composition of cohorts which, hence, require careful selection.

NGS protocols and bioinformatics pipelines, as well as microbial catalogues and culture collections, present with high degrees of biological and technical variability and, hence, approved standards are required to set a bar for which all data should be generated against. These standards should extend to sample collection, depletion of host cells and/or nucleic acids and extraction methods given the challenge of variable microbial loads at different environments (e.g. skin, wound, biopsy surfaces) (Amos et al., 2020). The need for validated NGS protocols, bioinformatics pipelines and microbial catalogues is huge to work with such sample kinds. Studies need to be sufficiently powered to allow statistically significant data interpretation and one needs to realize that markers such as skin lesion surface, bodily location, ethnicity, age of volunteer, (close) contacts with others and gender may be strongly confounding factors (Ruuskanen et al., 2022). Statistical analyses are essential and combined application of multiple validated tools is recommended. Visualization of the data supported by with alpha- and beta-diversity assessment and Principal Component Analyses (PCA) and Redundancy Analyses (RDA) will generate p-values that will withstand even the most critical evaluation. Effects caused by transient microbial contamination should be prevented or eliminated. This can be done by non-invasive surface rinsing or cleansing. *In vivo* studies should include sampling post last application of compounds. This will shed light on spontaneous microbiota recovery.

The international consensus seems to be that *in vivo* models are most important in assessing microbiota friendliness of cosmetics and therapeutics and, hence, provide a current method of choice. As a result, these services are beginning to become commercially available, for example Sequential Skin, a recent biotech, already offers large scale *in vivo* testing services (https://www.cosmeticsdesign-europe.com/Article/2022/03/03/microbiome-testing-startup-sequential-skin-opens-up-b2b-in-vivo-service-sequential-bio).

## Microbiome friendly label

Defining skin microbiome friendliness essentially equals the assessment of the safety of skin actives in relation to the absence or presence of microbiome perturbations. McBain et al. (2019) propose a 4-tiered framework for risk assessment and decision making. They refer to a 1). history of safe use, 2). reversible observations, 3). composition assessment and 4). searching for functional changes. We here focus on the composition assessment using a variety of approaches under a variety of environmental conditions and propose *in vitro*, *ex vivo* and *in vivo* opportunities (**Figure 1**). In practice, it is not very likely that all methods described in the above will be used and choices will be made based on for instance timing and budget available. A long period of prior safe use of compounds may also be an important testing format selection criterium. The methods promoted here are at best semi-quantitative. Additional and more precise, quantitative methods (e.g. species- or group-specific qPCR tests) may be needed at a certain stage.

**Figure 2:**
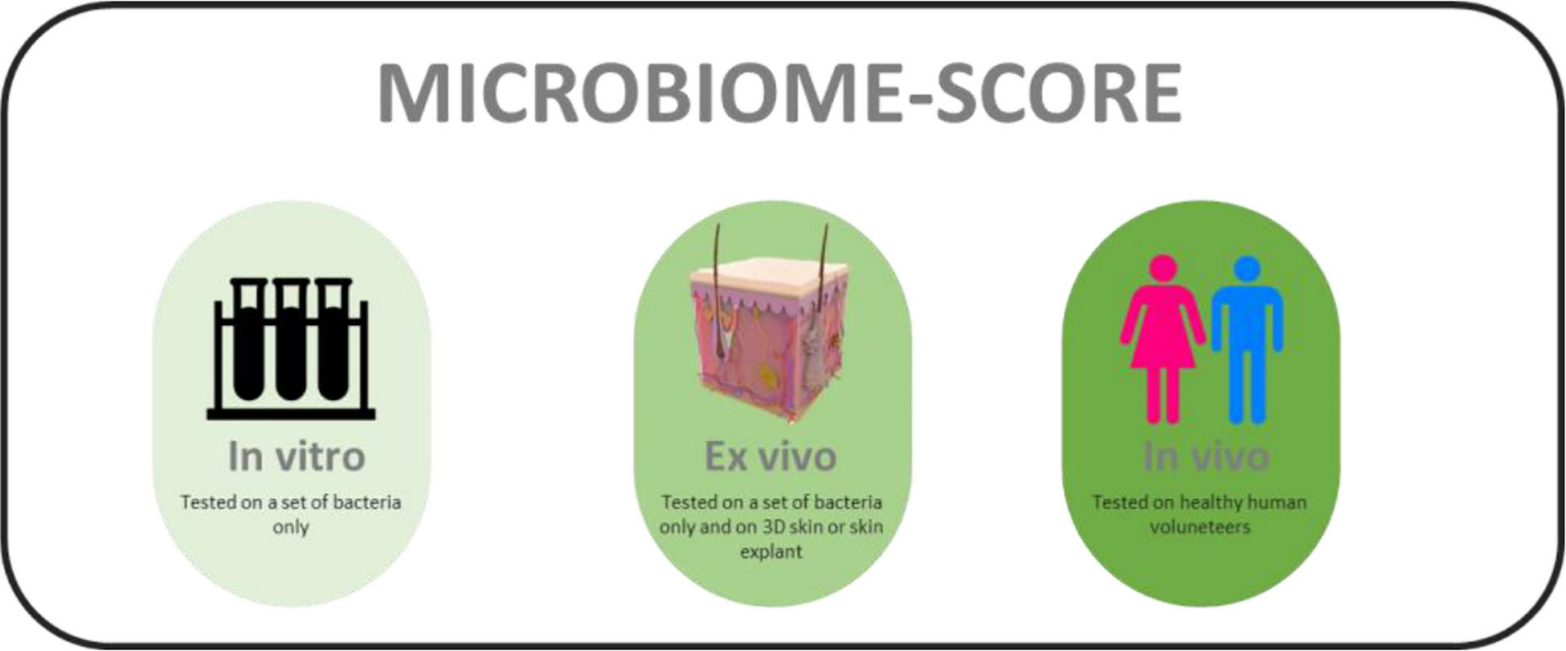
Hypothetical labels for defining testing methods used for assessing microbiome friendliness of a skin-directed product.

Reference materials (including carefully selected and product-appropriate negative controls) are important in standardization of any diagnostic microbiome application (Amos et al., 2020). Development of key reference materials could substantiate the value of quality labelling of (bio-)chemical compounds and final products. Still, PubMed screening with only three relevant key words (microbiome – friendly - skin) generates a mere 18 hits (search performed on the 17th of October, 2022). This implies that, despite the fact that there is a sizeable number of skin-targeted cosmetic and therapeutic products that carry a label of being microbiome friendly, science and regulatory bodies do not seem to take this concept very serious at the current stage (McBain et al., 2019). In order to properly address microbiome friendliness, a standardized and calibrated scientific approach is urgently required. We here propose that the definition of microbiome friendliness be based on serial *in vitro, ex vivo* and *in vivo* experimentation and be labelled as such. Labels should then adhere to the three categories where full “friendliness” validation can only be based on complete *in vivo* assessment. Anything less should be clearly defined on product labels and the term “microbiota friendliness” should be used in a restrictive and considerate manner. All three experimental regimens defined above have obvious value, including in consumer transparency, but the limitations associated with, for example, working *in vitro* only should be made clearly visible. It might be useful to define a microbiome scaling score that would help to define how a product has been tested rather than simply stating that a system has been tested one way or another (**Figure 2**). This could be accompanied by a decision tree showing the exact experimental routing of the assessment procedure (Carvalho et al., 2022).

Claims to microbiota friendliness should be realistic and based on solid experimentation and facts. In 2021, the authors’ organization has conducted a small survey among 16 key opinion leaders (KOL) and scientists from the field about the scientific substantiation of “friendly” derma-care products and cosmetics. Twelve of the sixteen KOLs indicated that microbiome friendliness is an important quality label for the products. There is an industrial need for microbiome friendliness labeling but there should be more clarity on the type of label and how the proposed labeling can be useful. It should be transparent to customers what the value of the label is and how the attribution of a label is regulated. Customer benefit should be explained as well as the labeling itself. In general, the KOLs suggested that the scientific community should set a higher bar for ill-substantiated claims.

## Quantifying microbiome friendliness

All of the above is quite descriptive and qualitative in nature. It is important to define more precise thresholds upon which the effect of a compound or formulation can be quantified rather than qualified as microbiome friendly or not. In case of *in vitro* testing does a ^1^log drop in CFU count imply that a product is still microbiome friendly or not? Describing a more precise quantitative NGS-based threshold is even more complicated involving species-specific read quantification. There are also biological variables to take into account: do we, for instance, allow for the microbiota to adapt to compounds or formulations or do we only screen for immediate effects? For practical and statistical interpretation of claims for microbiome-friendliness, defining the threshold of microbiome balancing and/or friendliness as the state where a compound or formulation does not unproportionally affect the growth of any resident and detectable microbial species does seem reasonable. In the mutual presence of at least two microbial species overall only a small change of the individual shares of the said microbes in the total number of the respective group of microbes is observed upon comparison with an appropriate control. A small change in this context is less than ±75%, in particular not more than +75% and not less than -25%, after 4h of incubation at 37°C. It is not unusual that other ingredients lead to significantly higher changes, i.e. changes of more than ±100% or even more than ±500% or even result in the disappearance of one or more of the microbes. These are early stage suggestions that require further international acceptation and standardization. We here have limited ourselves to bacterial microbiota not including important viruses, parasites and molds (Ruuskanen et al., 2022). We propose that *in vitro and ex vivo* analyses only provide basic knowledge and that such findings need to be substantiated by *in vivo* data. Only all-encompassing datasets can be used to define effective microbiome friendliness and labelling compounds or commercial products as microbiome friendly should be performed with care; claims should always be accompanied by a description of the analytical studies performed for the compound in question. Recently, the analysis of the skin metabolome has been suggested as a supplementary tool of importance (Emmert et al., 2021; Misra et al., 2021; Roux et al., 2022).

## Conclusion

The commercial offering for microbiome friendly labeling is already diverse. At MyMicrobiome (www.microbiome-friendly.com) a compound-specific certification can be obtained. The certificates obtained are based on *in vitro* generated data only. The single current *ex vivo* skin testing laboratory (Labskin, www.labskin.co.uk) has developed a co-cultivation system to test cosmetic ingredients for their microbiota friendliness. Proderm (www.proderm.de) and BaseClear (www.baseclear.com) have developed a label that is based upon NGS-based sequencing after *in vivo* methods. Straticell (www.straticell.com) has developed proprietary transcriptomics databases that can define skin microbiota changes. Many of the big pharma and cosmetic companies perform detailed microbiota research within their proper facilities. How this is used for defining microbiota friendliness is not always transparent. We have to conclude that we are still far away from a Gold Standard approach that reliably and universally defines microbiome friendliness to a level where regulatory bodies such as the US Food and Drug Administration (FDA) can start validating and accepting claims. To date, the accreditation currently offered is not backed up by any institution other than the companies awarding them; this should change. Changes in the microbiota that improve their quality (e.g. elimination of pathogens) are eagerly awaited to potentially help to fill the wavering global antibiotic development pipelines. Single bacterial cell approaches should be developed as well. Adequate microbiome friendliness assessment procedures will help in defining next-generation precision skin beauty and care products and therapies (Bouslimani et al., 2019). Employing the proposed approach and structure and performing the underpinning science in a transparent and science-led format will help to build trust with consumers. This sector still is in relative infancy and ahead of its predicted growth in the coming years. It will also be applicable to other areas of human healthcare where microbiome friendly claims may be applicable in the future.

## Data availability statement

The original contributions presented in the study are included in the article, further inquiries can be directed to the corresponding author/s.

## Author contributions

All authors listed have made a substantial, direct, and intellectual contribution to the work and approved it for publication.
